# If I Can Live

**Published:** 1910-01

**Authors:** 


### If I Can Live.

If I can live
To make some pale face brighter and to give
A second luster to some tear-dimmed eye,
Or e'en impart
One throb of comfort to an aching heart,
Or cheer some wayworn soul in passing by;

If I can lend
A strong hand to the fallen or defend
The right against a single envious strain,
My life though bare
Perhaps of much that seemeth dear and fair
To us of earth, will not have been in vain.

The purest joy,
Most near heaven, far from earth's alloy,
Is bidding cloud give way to sun and shine;
And 'twill be well
If on that day of days the angels tell
Of me, "She did her best for one of Thine."

—*Selected.*

Rectal palpation is an essential part of the examination in most acute intra-abdominal affections.—*American Journal of Surgery.*

---

Remorse is the form that failure takes when it has made a grab and got nothing.—*The Philistine.*

